# A Semantic-Based Cognitive Training Programme on Everyday Activities: A Feasibility and Acceptability Study among Cognitively Healthy Older Adults

**DOI:** 10.1155/2023/2153223

**Published:** 2023-08-23

**Authors:** Nikki Tulliani, Rosalind Bye, Michelle Bissett, Samantha Coutts, Karen P. Y. Liu

**Affiliations:** ^1^School of Health Sciences, Western Sydney University, Penrith, NSW, Australia; ^2^Faculty of Health, Southern Cross University, Gold Coast, QLD, Australia; ^3^Department of Rehabilitation Sciences, The Hong Kong Polytechnic University, Hong Kong

## Abstract

**Background:**

During the normal ageing process, a person's cognitive functions and memory gradually decline, which can affect their ability to perform everyday activities including cooking, cleaning, managing finances, and shopping. Semantic memory encoding strategies benefit older adults' cognitive and functional performance. Such strategies can be taught by an accessible, cost-effective, and flexible app-based programme. Currently, no studies examine such an app-based programme focussed on everyday activities.

**Objectives:**

To determine if an app-based programme constructed on the principles of semantic memory encoding strategies, targeted towards older adults, called Enhancing Memory in Daily Life (E-MinD Life) is (1) feasible by examining acceptance, engagement, and attendance and (2) acceptable by examining the perceived effectiveness, relevancy, clarity, and convenience.

**Methods:**

Eleven participants were recruited to a nine-week (18 sessions) programme using E-MinD Life. Feasibility was measured by collecting data on recruitment and retention rates, attendance, and duration of sessions. Acceptability was measured via a Likert scale questionnaire and free comments. Likert scale responses were analysed using descriptive statistics; open-ended responses were categorised qualitatively via constant comparative approach.

**Results:**

Nine participants completed the programme. Overall, most participants found the programme relevant, convenient, logical, and easy to understand and perceived it to be effective to address functional cognitive problems impacting performance of everyday activities. The results from the qualitative analysis showed that participants found the programme enjoyable and the interaction with the research team throughout the intervention beneficial.

**Conclusion:**

E-MinD Life shows promise as the focus of further research to determine the effectiveness of the programme and sematic-based cognitive strategies in maintaining cognition and performance in everyday activities among older adults with and without cognitive impairment.

## 1. Introduction

“Have you seen my car keys?” “Have I taken my medication today?” “Did I close the front door when we left the house?” “Did I turn the iron off?” Subjective memory complaints, often involving everyday information such as in these examples, are commonly expressed by cognitively healthy older adults [1–4]. Numerous studies have identified a link between the normal ageing process and a decline in memory [5–7]. Age-associated memory decline, related to healthy ageing, refers to modifications in the brain that impact cognition but have no apparent neuropathological cause [8]. However, in recent years, mild cognitive impairment (MCI) has received much attention in research and clinical fields relating to ageing and in particular dementia and associated disorders. MCI is positively linked with ageing and can be defined as a decline in cognition greater than that commonly expected for an individual of a similar age and education level [9]. Importantly, it has been proposed that MCI can signal the beginning of the neuropathological changes associated with dementia, gradually progressing over many years prior to a clinical diagnosis of dementia [9, 10].

Memory decline is associated with a decline in performance of instrumental activities of daily living (IADL) such as completing household chores, shopping, and managing finances [11]. Numerous research studies have highlighted the effect that the decline in cognition associated with MCI and dementia has on an individual's ability to independently perform IADL, even from early stages of the disease [11–14]. Losses in functional autonomy have been found to not only reduce individuals living with MCI and dementia's quality of life [15] but have also been shown to contribute significantly to family and carer burden [16, 17]. Therefore, early intervention targeted at memory decline is vital to prevent decline in everyday functioning and facilitate community living as well as to promote well-being and healthy ageing for healthy older adults and older adults in the preclinical stage of MCI and dementia.

All IADL require successful encoding, consolidation/storage, and retrieval of information [18]. Appropriate encoding of information is essential for successful retrieval [19]. Interventions targeted at the later stages of memory (storage or retrieval) may assist an individual with recalling previously stored information; however, they will not assist the individual to retain or recall new information. Individuals who inadequately encode information in the first place cannot be assisted by such interventions [20]. As daily living revolves around our ability to carry out IADL, it is therefore important to address how we encode and remember information about performing IADL and whether age-associated declines in these processes can be delayed, offset, or even improved.

Although research indicates that individuals with MCI may display deficits in all stages of memory [21], it has been demonstrated that the majority of the memory-related cerebral network changes in individuals with MCI occur during the encoding phase [20]. It has also been identified that encoding is the least effective memory stage in people with dementia [22]. The inability to retrieve encoded information may be a contributing factor to the reduction in functional performance in IADLs often associated with ageing and particularly in individuals with MCI and dementia. This may be due to their inability to organise and sequence the information required to be interrupted and actioned. Therefore, this study focuses on trialling a programme addressing the encoding stage of memory in older adults, teaching new encoding strategies as a preventive measure against memory loss in later life.

During the encoding stage of memory, elaborate encoding strategies that have been shown to improve memory performance [19]. There are three fundamental ways in which information can be encoded—visually, acoustically, or semantically. Visual or perceptual encoding is the process of capturing and storing visual information in memory, including images and objects, in a format that enables later retrieval [23]. Acoustic or auditory memory encoding is the process of capturing and storing auditory information in memory. It involves encoding sound waves or auditory stimuli in a manner that facilitates retention and later retrieval [24]. Semantic encoding *describes* the process that involves converting new information to memory by focussing on the meaning of the concept and word associations [25]. For instance, as an illustration, one approach may involve an individual mentally visualising themselves or others engaging in an activity, after watching a video depicting that activity (perceptual encoding). Alternatively, they can receive auditory information detailing the steps and context of the activity and use verbal repetition of the steps or the incorporation of the steps into a song or rhythm (auditory encoding). Another option is to read the sequential steps of the activity and make word-based associations from the presented information (semantic encoding). Considering the visual and auditory changes associated with ageing, semantic encoding techniques are a suitable choice for encoding memories among older adults. These techniques focus on the meaning and conceptual understanding of information, which can compensate for age-related declines in visual and auditory processing abilities [26].

Semantic memory, also called conceptual knowledge or declarative memory, is a type of long-term memory, which contains general knowledge about objects, word meanings, facts and concepts, rules, and people, and is acquired through our experiences of the world [27–29]. Semantic memory stores information by organising general knowledge into networks of connected ideas or concepts (schemata), and this helps us relate new information (episodic memory) to what we already know (semantic memory) [29]. This association allows us to retrieve information more efficiently, as semantically related objects are processed at faster rates than unrelated objects [19].

Semantic memory strategies include techniques such as semantic priming, picture naming, semantic fluency, conceptual hierarchies, and chunking association. These techniques have shown promising results for enhancing memory performance in healthy older adults [30–34], older adults with MCI [32, 34, 35], and older adults with dementia [32, 33].

Strategies that can be paired with semantic techniques to further enhance encoding include self-generation and the production effect. Self-generation is an encoding strategy using semantic memory processes where a person generates their own words, concepts, or items to enhance learning and memory of information rather than simply reading words, concepts, or items provided to them [36–39]. This strategy has been shown to improve the initial acquisition and learning stage of memory (i.e., the encoding stage) as well as the final stage of memory where information is retrieved/recalled [37, 40]. Numerous studies have shown the benefits of self-generation on memory performance among healthy older adults [38, 41, 42]. The production effect is the result of simply producing or reading information aloud, rather than silently. It has been shown that reading aloud can improve recognition memory by up to 20% [43].

Many of the described techniques are interrelated, and similarities can be seen across the different techniques. In this study, we developed a memory encoding intervention programme utilising semantic-based cognitive techniques, alongside self-generation, and the production effect.

Like most countries, Australia is facing a rapid increase in the number and percentage of older adults, with numbers expected to continually to grow [44]. By 2066, it is estimated that older adults will make up between 21% and 23% of the Australian population [44]. As the proportion of older adults increases, so too does the prevalence of MCI and dementia with the number of people living with dementia in Australia alone predicted to reach over 550,000 by 2030 [45]. A considerable proportion of people living with MCI and dementia lives in the community. In 2021, two in three people in Australia with a diagnosis of dementia were living at home in the community [46].

Due to the high proportion of people with dementia living in the community, there is a need for increased research into community-based interventions [47]. At home, intervention is an essential part of cognitive rehabilitation programmes as consumer access and funding can often place limitations on administration of such interventions by healthcare professionals [48]. However, individuals with memory impairment may have difficulties with self-administration of traditional cognitive intervention as they may struggle with self-correcting errors or remembering the steps to the task [49]. Computer-based programmes present an opportunity to potentially enhance and extend the effect of cognitive intervention programmes beyond professional-led training. A systematic review [50] examining the effectiveness of computer-based intervention on cognitive performance among individuals with MCI concluded that computer-based interventions had three key advantages in comparison to traditional paper-based interventions. Firstly, a computer-based intervention allows tailoring of the programme based on the individual's neuropsychological needs. Secondly, computer-based programmes have the potential to provide real-time feedback to both the user and the clinician. Thirdly, computer-based programmes allow for interventions to be available to a greater population; traditional interventions are not accessible on a large scale and therefore do not reach the needs of the increasing ageing population [50]. Finally computer-based programmes provide a cost-effective, self-administered, and time flexible means for individualised interventions under the guidance of a rehabilitation professional; traditional cognitive training and rehabilitation tend to rely on group intervention programmes, requiring individuals to travel to a designated location to receive intervention [50].

Considering that the Enhancing Memory in Daily Life (E-MinD Life) programme is in its early stages of development, it is vital to identify issues pertaining to its processes, feasibility, and acceptability before assessing its clinical outcomes [51, 52]. As a preliminary measure, before subjecting older adults with MCI or early dementia to the testing of E-MinD Life, it is crucial to gather feedback about the programme from cognitively healthy older adults. This group is best positioned to provide valuable insights and essential quality feedback, which can then be utilised to make necessary improvements prior to conducting future studies with an older population affected by cognitive impairment. Therefore, the aim of this study was to determine (1) the feasibility of self-administering an app-based, and everyday activity-focussed, cognitive strategy programme constructed on the principles of semantic memory called Enhancing Memory in Daily Life (E-MinD Life) by examining cognitively healthy older adults' engagement with the programme and (2) the acceptability of the everyday activity-focussed E-MinD Life by examining the perceived effectiveness, relevancy, clarity, and convenience of the programme among cognitively healthy older adults.

## 2. Materials and Methods

### 2.1. Participants

This study was part of a larger study examining different types of memory encoding strategies (perceptual-based and semantic-based). This current study reports on the feasibility findings from the semantic-based cognitive programme. As this was a newly developed intervention programme, identifying any key issues relating to its processes and feasibility is essential prior to evaluating its clinical effectiveness [51, 52]. Obtaining the perspectives from healthy older adults was an essential initial step in examining the feasibility of this proposed intervention prior to any attempt to administer the programme with a clinical sample of older adults with MCI.

Participants were recruited via convenience sampling at two independent living retirement villages in Sydney, Australia. A recruitment presentation about the study was given by one of the researchers (NT) at a “resident meeting,” and recruitment flyers and information sheets were handed out. English-speaking adults aged 65 years or older, who showed an interest in the programme and self-reported to be cognitively healthy with no previous psychiatric or memory disorder history or other neurological illnesses that may affect their cognition, were eligible to participate. Potential participants contacted the researcher (NT) to express interest in the study and were subsequently visited in person by the researcher (NT) and screened for inclusion in the study. Potential participants were excluded from participating in the study if they scored (1) less than 24 on the Mini-Mental State Examination, 2^nd^ edition, standard version (MMSE-2:SV-2:SV) [53], or (2) 5 or more on the 15-item Geriatric Depression Scale (GDS-15) [54]. Participants were not required to have any previous experience with an app-based device, any level of computer literacy, or access to the Internet.

Approval to conduct the study was obtained from the Human Ethics Research Committee at Western Sydney University, Australia. All participants provided written informed consent prior to commencing the study.

### 2.2. Intervention

The everyday activity-focussed intervention was designed on semantic encoding principles. Semantic encoding techniques were applied to 12 common IADLs essential for independent community living and built into E-MinD Life on the app-based platform, Qualtrics (http://www.qualtrics.com).

The semantic encoding techniques used in E-MinD Life were developed with reference to the previous studies describing semantic priming, picture naming, *clustering,* category fluency, conceptual hierarchies*, self-generation,* chunking association, and honeycomb techniques [34, 35]. The application of these techniques is outlined below:

*Semantic priming*: the participant is first shown a single word relating to an IADL, called the “prime” (e.g., cup). This is followed by a short delay before a target word is placed over the top of the prime (e.g., tea). The participant then decides whether the two words are related or unrelated.

*Picture naming*: the participant is presented with an image relating to an IADL (e.g., a photo of a tea bag or a photo of a person removing a tea bag from a cup by squeezing the tea bag against a teaspoon). The participant is then asked “what is this item and what is it used for?” or “what is happening in this image?”.

*Category fluency (clustering)*: the participant is given word cards relating to the IADL (e.g., morning, kitchen, husband/wife, and kettle); the participant is then asked to sort them into four categories: person, place, time, and object.

*Conceptual hierarchy*: the participant defines a sequence of mappings (creates a hierarchy) using a selection of jumbled words related to the IADL (e.g., 2-minute brewing, morning tea, afternoon tea, breakfast, time, hot beverage, teabag, fridge, milk, coffee, and sugar). A main concept is given for the group of jumbled words (e.g., hot beverage); then, out of these words, subconcepts are determined (e.g., time, cupboard, and fridge), and examples of these subconcepts (e.g., sugar, coffee, and teabags) all belong to the subconcept cupboard, whilst 2-minute brewing, morning tea, and afternoon tea all belong to the subconcept time. Once the participant had completed a hierarchy, they were asked to examine it to ensure it was properly arranged and prompted by the therapist if rearrangement was needed.

*Chunking association*: each IADL was broken down into six steps based on activity analysis and task breakdown. Participants were provided with these steps in word format and encouraged to learn and remember the instructions of the IADL by reading them out loud (*production effect) and* chunking the information into steps. Participants were encouraged to use verbal repetition during this task to further enhance working memory and learning [55]. The honeycomb technique [35] was used in conjunction with chunking by giving contexts to the chunks of information. This concept allows the steps of the IADL to form a story. The participant relates the information according in relation to four categories: person, place, time, and object (or problem/solution) and then verbalises their story.

*Self-generation and production*: in the self-generated task, the six steps were presented with at least one word missing from the step as indicated by a blank line. Participants were asked to fill in the blank with the most logical choice. For example, if a participant was working on the meal preparation activity of “making a hot beverage,” they would be presented with a step such as “pour the _____ water into the cup” or “use the _____ to stir in the _____ and _____” and the participant would be required to generate and read aloud to produce an appropriate response to complete the step (e.g., boiling or hot, spoon or teaspoon, milk or cream, and sugar or sweetener). As participants progressed through the programme, additional words were removed from the steps until participants were required to verbalise the entire step without cueing.

The daily activities were chosen based on the results of a previous study examining the most important, frequently performed, and cognitively demanding IADL completed by older adults that facilitate independent community living [56]. The 12 IADL were divided into three blocks, each containing four activities. These blocks could be broadly classified under “meal preparation and clean-up activities” (e.g., making a hot beverage, making a hot breakfast, and preparing a salad), “laundry activities” (e.g., washing clothes, ironing clothes, and packing away laundry), and “communication and community activities” (e.g., making and attending medical appointments, grocery shopping, and paying bills). Each block of IADL was designed to be the focus of a three-week semantic-based cognitive strategy learning period, with the total programme length being nine weeks.

### 2.3. Procedure and Measures

After obtaining the informed written consent, the participants received the nine-week E-MinD Life programme. The programme itself consisted of three 60-minute sessions per week: two self-administered sessions and one face-to-face individualised therapist-led session. Twelve weeks was allocated to provide participants with some degree of flexibility to complete a nine-week programme. In support of the sessions, participants were provided iPads with the semantic strategy IADL programme installed. The weekly therapist-led sessions were individualised and tailored according to the participants' needs and challenges faced. These sessions were conducted at the participants' home and facilitated by a research assistant with a background in occupational therapy. During these sessions, the semantic-based cognitive strategies utilised in the programme were taught to the participant using paper-based activities, followed by using the iPad to complete the scheduled session. Participants were provided with instructions on how to operate the iPad including information on how to turn the iPad on/off, how to charge the iPad, how to set reminders, and how to access the programme. After the therapist-led session, the participant scheduled two days that week to independently complete the programme by completing the iPad activities practised during the therapist-led session.

#### 2.3.1. Baseline Characteristics and Demographics

Prior to the commencement of the programme, demographic information such as age, gender, educational level, living arrangements, and current occupation was obtained from the participants through interview by the primary researcher (NT). The Lawton Instrumental Activity of Daily Living Scale [57] was administered to assess independent living skills. Demographic characteristics of participants were summarised by using mean and standard deviation for continuous variables and number and percent for categorical variables.

#### 2.3.2. Feasibility of E-MinD Life

*(1) Feasibility: Recruitment and Retention Rate*. Recruitment success was defined as 10% of potential participants agreeing to be enrolled in the study. Based on a meta-analysis of computerised cognitive training among older adults with MCI which reported dropout rates ranging from 0 to 32% [58] and a meta-analysis of cognitive interventions among older adults with MCI reporting dropout rates as much as 36% [59], a successful retention rate was defined to be less than 20% attrition of participants who commenced the nine-week programme.

*(2) Feasibility: Attendance and Completion Rate of Therapist-Led and Self-Administered Sessions*. Attendance at the nine-weekly therapist-led sessions was recorded, and a percentage attendance rate was calculated. Scores of 1, 0.5, and 0 were given for each “completed,” “partially completed,” or “not attempted/incorrect session completed” self-administered session, respectively. A participant completing at least one activity whilst failing to complete the entire session was given a “partially completed” score of 0.5. A participant failing to appropriately select the right session numbers whilst in the programme obtained a session score of 0 for “incorrect session completed.” Participant completion rate was calculated by adding up the scores and dividing by 18, with 18 being the highest possible score.

*(3) Feasibility: Barriers to the Implementation of the Programme*. Reasons for not completing programme sessions were recorded by the research assistant implementing the programme.

*(4) Feasibility: Time Taken to Complete the Self-Administered Sessions*. The time taken for each participant to complete the 18 self-administered sessions was recorded, and an average duration was calculated for all participants. If the session was partially completed, the duration was omitted from the calculation.

#### 2.3.3. Acceptability of E-MinD Life

An acceptability questionnaire was designed by the primary researcher (NT) and then revised by the research team. The questionnaire design was informed based on a previous study [60] and targeted four attributes: (1) perceived effectiveness—how participants perceived the intervention in allowing them to acquire, apply, and master the semantic-based cognitive strategies; (2) relevancy—if the daily activities were relevant and programme suitable for use among older adults; (3) convenience—perception of how easy it was to complete the intervention; and (4) clarity—if the programme was logical and easy to follow, including if instructions were detailed enough to complete the intervention independently. The questionnaire covered four important areas of the programme including (1) overall programme, (2) therapist-led sessions, (3) self-administered sessions, and (4) the computer programme and iPad use. The questionnaire was piloted with colleagues of the research team. The questionnaire consisted of 41 questions. Examples of questions included the following:

1. I feel the program would be useful in maintaining the ability to perform daily tasks for people with memory difficulties (perceived effectiveness)
2. During the program I found that I used the strategies taught (semantic technique) when carrying out other daily activities in my day (perceived effectiveness)
3. The daily activities in the program were relevant to me (relevancy)
4. The program using the memory strategy to enhance remembering of the activity steps is relevant to me (relevancy)
5. Using an iPad was a convenient approach to completing the program (convenience)
6. The program schedule was suitable for me (3 times per week) (convenience)
7. I felt one session a week with the therapist was sufficient to aid my understanding of the strategy (semantic technique) (clarity)
8. I felt the program was easy to navigate (clarity)

Participants rated questions on a four-point Likert scale ranging from completely disagree to completely agree. Higher scores indicate that the participant perceives the programme to be effective, relevant, suitable, and convenient. Participants were encouraged to comment freely [61] and provide suggestions in relation to each of the four attributes, self-administered sessions, therapist-led sessions, and the use of a computer-based program. Participants were also asked (1) if they would recommend the programme to other older adults 65 years and older, (2) if they would use the programme as part of an occupational therapy intervention and be satisfied with the therapy provided, and (3) what were the most enjoyable and most challenging parts of the programme.

Each participant was provided with the questionnaire at the completion of the nine-week E-MinD Life programme. Participants were given a one-week period to complete the survey prior to collection by one of the researchers (NT) not directly involved in the delivery of the intervention.

### 2.4. Data Analysis

#### 2.4.1. Participant Demographics and Baseline Characteristics

Demographic and baseline characteristics of participants were summarised by using mean and standard deviation for continuous variables and number and percent for categorical variables.

#### 2.4.2. Feasibility of E-MinD Life

Data were analysed using Microsoft Excel to produce summary statistics. Participant recruitment and retention rates, attendance to the programme, duration of sessions, and barriers to the implementation of the programme data were summarised and reported as frequencies and proportions or as free text.

#### 2.4.3. Acceptability of E-MinD Life

Data were analysed using Microsoft Excel to produce summary statistics. Likert scale responses were grouped into “broad agreement” (comprising agree (3) and completely agree (4)) or “broad disagreement” (comprising fair (2) and poor (1)) and presented in graphical form in Section 3.3.1. Each attribute (perceived effectiveness, relevance, convenience, and clarity) was averaged for each of the four areas (overall programme, therapist-led sessions, self-administered sessions, and the computer programme and iPad use) by calculating the sum of percentage agreement scores of all question with a participant given ratings of 3 or 4 and then dividing that amount by the total number of participants. Attributes that scored an agreement level of >79% were deemed acceptable, 70-79% questionable, and <70% unacceptable [62].

Qualitative analysis, specifically open coding to group like responses [63, 64], was used to categorise free text responses. The qualitative findings provided insight into the experiences of older adults during the nine-week E-MinD Life programme. Two overarching groupings emerged: (1) the most enjoyable parts of E-Mind Life and (2) programme challenges and suggestions for future iterations of E-MinD Life [63, 64]. Under each grouping, categories and subcategories were identified [63, 64]. Under supervision from the second author (RB), the first author (NT) coded each open-ended question's responses. Provisional categories were developed by identifying similarities and differences between participant survey responses [63, 64]. The research team (NT, BR, KL), once consensus was reached, amended and renamed titles of categories and subcategories.

## 3. Results

### 3.1. Participants: Baseline Characteristics and Demographics

Nine participants completed the study. Participants ranged in age from 72 to 85 years with three males and six females being recruited. GDS-15 scores ranged between 0 and 4 indicating no signs of depression. All participants' MMSE-2:SV scores were within the “normal cognition” range. The Lawton IADL scores indicted that the participants were independent in most everyday activities. Participant demographics and baseline characteristics are described in Table 1.

### 3.2. Feasibility of E-MinD Life

#### 3.2.1. Feasibility: Recruitment and Retention

Of the 54 older people who attended the information sessions across the two independent living retirement villages, 23 agreed to participate in the study. Three of these people did not meet the selection criteria based on MMSE-2:SV score. After excluding the three participants who did not meet the inclusion criteria, the recruitment rate was 20.37%. Eleven participants were allocated to this study and all consented to participate. The remaining participants were allocated to another study outside the scope of this paper. There were two dropouts over the duration of the study (retention *rate* = 81.81%). One participant withdrew from the study due to a health-related issue, and one participant died during the intervention period.

#### 3.2.2. Feasibility: Attendance and Completion Rate

Participants' attendance at the therapist-administered sessions ranged from 5 (55.56%) to 9 (100%) sessions. Seven participants (77.78%) attended all nine sessions. The average number of sessions attended was 8.44 (93.78%) (*SD* = 1.33). Reasons provided for nonattendance or rescheduling of the therapist-led sessions included medical appointments, travel and holidays, illness, or unwell spouse.

Participants' attempt at the self-administered sessions ranged from 6 (33.33%) to 25 (138.89%) sessions over the nine-week programme. The average number of sessions attempted was 18.11 (*SD* = 5.09) out of the 18 scheduled sessions. All nine participants completed at least one extra session (i.e., they repeated a session they had already completed). The average number of extra sessions completed was 3.33 (*SD* = 2.12). One participant (11.11%) attempted and completed all 18 sessions.

Reasons provided for noncompletion of the self-administered sessions included difficulties with the iPad, technical difficulties with the programme, being unwell, or other commitments including visitors and holidays.

#### 3.2.3. Feasibility: Duration

**Figure 1:**
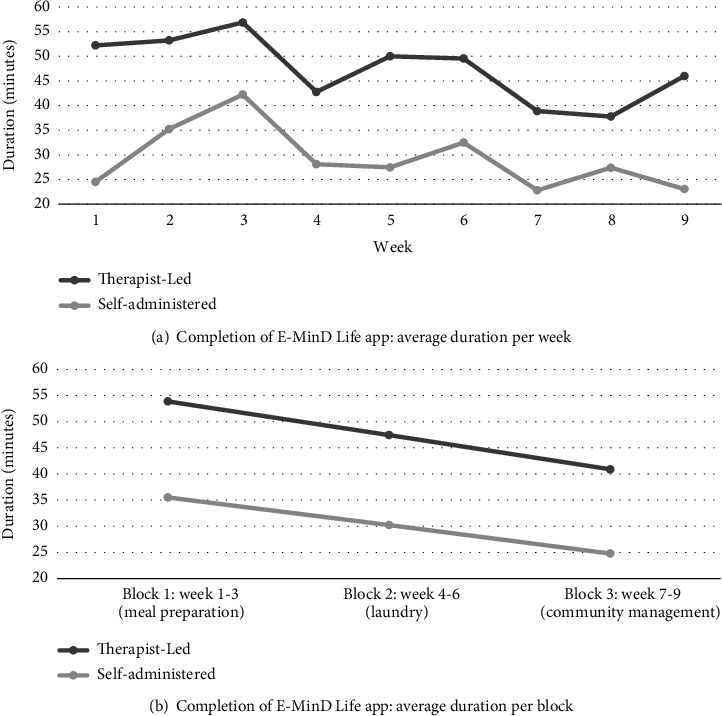
(a, b) Time taken to complete E-MinD Life programme.

The average time for the E-MinD Life programme during the therapist-administered sessions was 47 minutes (*SD* = 16.46), ranging from 25 to 101 minutes. The therapist-led sessions increased in duration from session 1 to session 3 of each block (Figure 1(a)), consistent with the increasing difficulty of the programme. The average duration of the therapist-led sessions reduced over the course of the programme from an average of 53 minutes (*SD* = 20.12) for block one, 47 minutes (*SD* = 14.96) for block two, and 40 minutes (*SD* = 11.44) for block three (Figure 1(b)).

Self-administered sessions ranged from 12 to 95 minutes. The average time for the self-administered sessions was 29 minutes (*SD* = 18.10). The self-administered sessions increased in duration from session 1 to session 3 of block one and two (Figure 1(a)), consistent with the increasing difficulty of the programme. However, block 3 saw an increase in duration between the first and second session and then a drop in duration for the final session of the block/programme. The average duration reduced over the course of the programme from an average of 35 minutes (*SD* = 24.44) for block one, 30 minutes (*SD* = 15.28) for block two, and 24 minutes (*SD* = 9.86) for block three (Figure 1(b)).

### 3.3. Acceptability of E-MinD Life

#### 3.3.1. Acceptability: Quantitative Results

**Figure 2:**
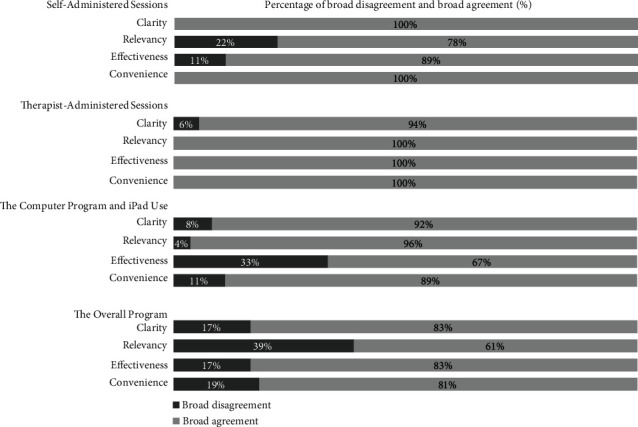
Older adults' acceptability of the E-MinD Life programme.

At the completion of the nine-week programme, participants indicated broad agreement across all three dimensions of feasibility, clarity, and relevancy. Most participants (83%) perceived the overall programme to be effective in allowing them to acquire, practise, and master the semantic-based cognitive strategies. Sixty-one percent of the responses indicted that participants rated the overall programme to be appropriate for older adults and thought that the daily activities were applicable. Eighty-one percent of responses showed broad agreement that the overall programme was easy to participate in. Eighty-three percent of responses noted that the overall programme was logical and easy to understand. The results for the four dimensions (perceived effectiveness, relevancy, convenience, and clarity) across the four areas (overall programme, therapist-led sessions, self-administered sessions, and the computer programme and iPad use) are presented in Figure 2.

Of the nine participants, 88.9% (*n* = 8) rated broad agreement when asked if they would use E-MinD Life if it was part of an occupational therapy intervention and would be satisfied with the therapy provided. All participants (*n* = 9) rated broad agreement when asked if they would recommend the programme to other older adults aged 65 years and older.

#### 3.3.2. Acceptability: Qualitative Results

*(1) What Were the Most Enjoyable Parts of the Programme?*. Three overarching categories and 10 subcategories were generated from the qualitative data in relation to the grouping of most enjoyable parts of the programme. These categories are outlined in Table 2 with verbatim comments from the participants. In addition to these categories, several participants noted that their motivation to participate in the project was driven by their desire to assist others. Participants felt a sense of contribution to their community.

I enjoyed knowing that this is helping a programme that would keep people with some memory loss to have a better quality of life. (P7)

Hoping in some way to have been of some help in the research and to have been some help to others in the future. It was nice to meet the young people. (P3)

*(2) Programme Challenges and Suggestions for Future Iterations*. Two overarching categories and eight subcategories were generated from the qualitative data in relation to the grouping—challenges faced when completing the programme and suggestions for the future iterations of the programme. Results are outlined in Table 3 with verbatim comments from the participants.

## 4. Discussion

In this study, an everyday activity-focussed, semantic-based cognitive training programme called E-MinD Life was developed, and its feasibility and acceptability were evaluated. Overall, the results showed that the E-MinD Life programme, utilising semantic-based cognitive strategies, was feasible and acceptable among healthy older people. Several key findings were obtained.

Regarding the feasibility of E-MinD Life, although the number of participants in the study were few (*N* = 9), the authors deemed the recruitment rate (20.37%) to be successful as more than 10% of potential participants agreed to enroll in the study. This is similar to other studies examining recruitment and retention of older adults in health-based intervention studies. For example, Jancey et al. [65] considered a recruitment rate of 12.6% of their targeted sample to be successful for their intervention group. However, to improve recruitment rates, in future studies for E-MinD life, we will consider recruitment methods as reported by Forsat et al. [66] for older people clinical research. Recruitment methods that will be considered include the use of letters/mailings and use of newspapers that directly target the required population [66]. Our study only used letters/mailing and a promotion talk without advertising in newspapers. Furthermore, we will consider recruiting from a larger field by including referrals from primary care providers [67, 68] and geriatric assessment units [69] which are also reported to be effective for recruiting older adults for clinical research [66].

The retention rate in our study was high (81.81%) with only two dropouts over the duration of the study—less than 20% attrition. Consistent with retention strategies outlined to be effective by Forsat et al. [66], this study ensured the research staff conducting the intervention were consistent; return visits and sessions were prescheduled; and flexibility concerning the time and location of the intervention and data collection was applied. However, future studies of E-MinD life can consider monetary incentives to further improve retention rates [66].

Attendance to and completion of the programme suggest that the schedule of the E-MinD Life programme was feasible for older people. Joosten-Weyn Banningh et al. [70] considered attendance at 80% of sessions to be an appropriate adherence rate for older people with MCI enrolled in a cognitive behavioural intervention. Our study had an average attendance rate of 93.78% for the therapist-led sessions. Although the average attendance rate for the self-administered sessions exceeded 100%, this needs to be considered with caution as participants might incorrectly complete session beyond the scheduled 18 sessions. Future, iterations of E-MinD Life should include software to prevent participants from completing a scheduled session more than once. Although E-MinD Life consisted of sessions over nine weeks, the additional three weeks allowed for participant flexibility to complete the programme which may have led to better attendance rates than if the programme presented a more rigid structure. However, it needs to be acknowledged that all participants were motivated to either learn ways to address their cognitive concerns or assist in the development of an intervention to support memory decline. These factors may have positively influenced their willingness to attend and complete the programme.

Overall therapist-led sessions were substantially longer than self-administered sessions, with several possibilities to explain these findings. One of the strengths of E-MinD life was that no time limit was placed on the therapist-led session, allowing participants to take as long as needed to learn and practice the semantic-based cognitive strategy. Although each session of E-MinD Life was designed to take approximately 60 minutes, the average time taken for participants to complete self-administered sessions was only 29 minutes (*SD* = 18.10). This was compared to the average time of 53 minutes (*SD* = 20.12) taken during the therapist-led sessions. Participants may have familiarised themselves with content during therapist-led sessions, reducing the time it took to complete their own sessions. Therapist-led sessions may have also been longer due to the incidental social interaction during these sessions between therapist and participant. Alternatively, the “teaching approach” applied in the therapist-led sessions may have required additional time not needed during the self-administered sessions.

The time taken to complete the therapist-led sessions increased over the course of the programme, but the time taken to complete the self-administered sessions decreased. The increase seen in the therapist-led sessions was expected as the difficulty of the programme increased, and new activities were introduced as the programme progressed. It is unclear why the self-administered sessions became shorter. Several reasons may include participant familiarity with sessions over time, participants completing the tasks of the programme based on previous attempts (i.e., memorising the answers) without applying the taught semantic-based cognitive strategies, or boredom with the programme leading to less time spent in engagement with strategies. Future iterations of the programme will need to consider how to encourage participants to continue to apply the taught semantic-based cognitive strategies and only allow participants to progress from each task after an appropriate amount of time has been given to implement the semantic strategies.

Overall, the results indicate that older adults found the E-MinD Life to be an acceptable cognitive strategy programme.

With respect to the relevancy of the programme, all participants found the therapist-led sessions to be relevant for the teaching of the semantic-based cognitive strategies and initial learning of how to use the programme. However, only 61% of responses indicted that participants found the overall programme to be relevant. As all the participants are independent in IADLs, they may not see the need to have the memory encoding intervention at this stage in their life when they are healthy. A thematic analysis conducted by Tkatch et al. [71] examining older adults' perceptions of health, identified a key theme to be “maintaining day-to-day functioning is a priority for successful ageing.” This supports the relevancy of E-MinD Life despite only 61% of the responses indicating broad agreement. Furthermore, Tkatch et al.'s qualitative study [71] demonstrated that “health”-related interventions are needed for both healthy and unhealthy older adults to help them learn to adapt to health and functional changes as they age. One study [72] found that older adults were more likely to recognise and recommend follow-up for memory-related problems affecting others than if the same problems were affecting themselves. This may explain the findings regarding healthy participants acknowledging the relevancy of E-MinD Life in this study when they themselves were not experiencing memory problems. Future studies for E-MinD Life with healthy older adults may need to take an educational approach on the importance of memory-based and activity-focussed early interventions to aid successful ageing. Although 96% of responses indicted that participants found administering the programme via an iPad to be a relevant way of administering the programme, only 78% of responses showed that participants found the self-administered sessions to be relevant. The use of an app-based programme allows the programme to be self-administered, and the reasons why some participants did not find these sessions to be relevant need to be further explored. One study examining the acceptability of healthcare interventions [73] in older people found that people are more likely to adhere to intervention recommendations and have better clinical outcomes if they consider the intervention to be acceptable. Findings from our study require further exploration and consideration prior to any further development of the E-MinD Life programme. Older adults may disengage from the programme if they are unable to distinguish the relevancy to themselves. This finding needs to be considered when explaining the programme to participants in the future, emphasising the health promotion and preventative nature of the programme.

With regard to the perceived effectiveness of the programme, all participants (100%) found the therapist-led sessions to be effective in allowing them to acquire, apply, and master the semantic-based cognitive strategies in comparison to the self-administered sessions in which only 89% of responses indicated that participants found these to be effective. In regard to the app-based nature of the program, there were reduced reports of effectiveness, with only 67% of responses indicating that participants found the app to be an effective way of learning the semantic-based cognitive strategies. This result suggests that the app itself, rather than the content, may need some improvements to increase its perceived effectiveness. These results also highlight the benefit of a therapist being part of the intervention process. The perceived effectiveness of the programme reported by the participants may also be impacted by the quality and duration of the self-administered sessions, as discussed above. If not implemented as intended, the intervention programme may be considered to have low acceptability, thereby influencing its effectiveness [74].

Results from the qualitative analysis suggest that there are several strengths of the programme. Participants highlighted that one of the most enjoyable parts of programme was the ability to interact and connect with the research team implementing the intervention. This aligns with the increased duration of therapist-led sessions compared to the self-administered sessions. This finding should be considered when developing self-administered interventions, as incorporating an interactive component may enhance acceptability and feasibility outcomes. Although the programme was designed to be two-thirds self-administered, findings reveal that the therapist continued to play a valued role in the therapy process. This finding is similar to that from Piculell et al. [75] who found older adults required and preferred to have support when using mobile applications for health. Furthermore, participants in this study indicated that the therapist-led sessions allowed for easy clarification and the ability to ask questions about the programme when required. This is again consistent with the findings from Piculell et al. [75] where participants found it easier and quicker to ask for help rather than decipher the programme themselves.

Another important finding was that participants desired to engage with research to help others experiencing memory loss. This finding needs to be considered, as it may assist in the recruitment of participants in future studies. Alternatively, educating future older adults on the health promotion and preventative nature of the program may enhance engagement.

Results from the qualitative analysis suggest that minor changes to future iterations of the programme may further enhance its acceptability and usability. Regarding the challenges of and suggestions for E-MinD Life, some participants found the programme to be long and difficult to fit into everyday life. Some participants also found the programme to be too repetitive in nature. There were 18 sessions in total and the programme ran for 9-12 weeks. The number of training sessions was allocated according to a systematic review conducted by Jean et al. [76] which noted intervention periods consisting of between six and 20 to be the most cost-effective for research and clinical purposes. In addition, it was shown that those intervention periods greater than 12 weeks did not show a significant advantage over intervention lasting less than 12 weeks. Future iterations of the programme should consider the length of the programme to increase the adherence to the programme. Further research should review if a shorter programme consistent with Jean et al. [76] and less repetition is feasible, acceptable, and effective in maintaining cognition and functional performance.

Another challenge identified in the qualitative analysis was the difficulties with using the mobile application. Participants expressed that it was sometimes difficult to navigate the programme on the mobile application which resulted in errors. Difficulties with using the mobile application may explain why all participants completed more sessions than scheduled. This finding is consistent with a study examining the experiences of older adults with MCI using mobile applications for health [75] which found that participants had difficulties accessing app-based content and using the different functionalities available. However, several studies have highlighted the effectiveness of computer-based interventions for improving cognition among older adults, including those with cognitive impairments [77, 78]. Therefore, it is likely that future iterations of the programme need to consider the formatting of the mobile application including improvements to the visual design and visual prompts to enhance clarity and improve the user experience.

### 4.1. Limitations of the Study

This study has several limitations that could impact the generalisability of the feasibility and acceptability results. Firstly, the sample size was small and recruited from only two sites, thus limiting the generalization of results to a broader context. Secondly, only one app-based cognitive strategy programme was presented to the participants; therefore, it is not known how presenting more than one training programme option would affect a participant's ratings of the programme. Thirdly, although older adults with MCI are more in need of a programme like E-MinD Life, only healthy older adults were recruited in this study. Consequently, additional larger studies with a control programme group and participants with MCI are essential to determine the acceptability and feasibility of E-MinD Life among older adults with MCI. Furthermore, although this study was not designed to test intervention effect, participants did rate their perceived effectiveness. Finally, no specific outcome measures were administered to participants, and the ability to learn and apply the taught strategy was also self-reported by participants. Further studies are required to determine the effectiveness of the programme for improving cognitive functioning or IADL performance as well as determining the ability to learn and apply the semantic strategies.

### 4.2. What This Study Has Added

Findings from this study indicate that an everyday activity-focussed semantic-based cognitive intervention (E-MinD Life) administered via an app is feasible and acceptable to deliver to older adults as a health promotion programme to prevent the impact of possible cognitive changes.

## 5. Conclusion

Occupational therapy can support older adults to remain living in the community. These findings support the feasibility and acceptability of E-MinD Life, a combined instructor-led and self-administered semantic-based cognitive programme via an app-based platform as a health promotion programme for healthy older adults. Now that we have established the acceptability of E-MinD Life among healthy older adults and confirmed its feasibility within this population, further research is required to determine the programme's feasibility and acceptability among older adults with MCI and mild dementia. Once feasibility and acceptability are explored with older adults with MCI and mild dementia, research can then be conducted to determine the effectiveness of the programme for maintaining of improving cognitive functioning and IADL performance in older adults with MCI and mild dementia.

### 5.1. Key Findings

1. E-MinD Life is an acceptable and feasible semantic-based cognitive intervention to administer to healthy older adults
2. The use of semantic-based cognitive strategies provides an opportunity to implement cognitive intervention at the encoding phase of memory
3. Future research is needed to evaluate the effectiveness of the programme in a wider sample size, including healthy older adults, as well as a sample of older adults with MCI or mild dementia

## Figures and Tables

**Table 1:** Participant characteristics.

	*n* = 9
Female (*n*, %)	6 (66.67)
Age (years, *mean* ± *SD*)	77.89 ± 4.51
Level of education	
Secondary education (*n*, %)	6 (66.67)
Higher education (tertiary—masters)	1 (11.11)
Higher education (tertiary—PhD)	1 (11.11)
Higher education (technical college)	1 (11.11)
Living arrangements (*n*, %)	
Living alone	2 (22.22)
Living with spouse	7 (77.78)
Current occupation (*n*, %)	
Retired and volunteering	5 (55.56)
Retired	4 (44.44)
GDS-15 (*mean* ± *SD*)	1.11 (1.17)
MMSE-2:SV (*mean* ± *SD*)	28.33 ± 2.61
Lawton IADL (*mean* ± *SD*)	7.89 ± 0.33

SD: standard deviation; GDS-15: 15-item Geriatric Depression Scale; MMSE-2:SV: Mini-Mental State Examination, 2^nd^ edition, standard version; IADL: instrumental activities of daily living.

**Table 2:** Summary of the participants' perspective on the most enjoyable parts of E-MinD Life.

What were the most enjoyable parts of the programme?		Participants' verbatim comments
Memory focussed	Increased awareness of memory in daily life	I became more aware of memory use in day-to-day things. (P7) Made me aware of what I need to re-learn. (participant no. 6)
	Stimulation to learn new skills and technology	Made me think and get brain cells functioning. (P6) It helped me sharpen my skills using a tablet (P5) The best part for me was using an iPad which I had not done before. (P2)
	Challenge at the right level	They became like a “game” to challenge me which I enjoyed. (P7) …really tested the brain with lots of different challenges. (P2) I found it took me a short time at each lesson to concentrate on what I was to do, to think differently. (P3)

The tasks	Systematic	Weekly visit from therapist. (P9) I felt that if you did these tasks regularly it made it easier to understand how to do the exercise. (P2)
	Practical	…really tested the brain with lots of different challenges and tests and also the practical everyday food preparation, ironing etc. (P2)
	Real life	I found the course very helpful, as well as enjoyable. I would strongly recommend it to other people in similar circumstances as myself. (P5) It was valuable in helping me interact with a younger generation, a skill which tends to be lost by my generation, living in an institution such as a retirement village. (P5) The exercises [IADLs] were many and varied. (P2)

Implementation and connection with programme team	Supportive approach	It was pleasant to have supervising examiners with cheerful attitudes. (P6) I appreciated learning how to use it [iPad] – Thanks to the people who helped! (P7)
	Clarifying aims, procedures, and resources	Therapist led sessions because any questions I had were clarified and I understood the aim/goal of the program. (P4) Good to get away from a screen and interact with therapist on task. (P2)
	Smooth operation	Interaction with co-ordinators and their good appointment keeping and interpersonal skills. (P7) There was a lot of organising done for this program and I congratulate those involved. (P2) I think I did better on my own because I did not feel stressed that I might be holding anyone up. (P3)

**Table 3:** Summary of the participants' perspective on the challenges when completing E-MinD Life and suggestions for future iterations to improve the programme.

Programme challenges and suggestions for future iterations		Participants' comments
Design	Reducing accidental/“working” errors	After explanation it was good but if any “working” problems occurred I found that challenging. (P7) … while I was on holiday, I messed up by opening a therapist led part of the program. (participant no. 4) … if you press the wrong button, you cannot remove the dot, so might show an incorrect error when all you did was accidently pressed the wrong line button. (P2)
	Orientation to session	I found it took me a short time at each lesson to concentrate on what I was to do, to think differently. It was stressful for me as I like to get things right. (P3)
	Task relevancy	Depend on the level of education of the people using “word” based activities. (P4) Relevancy! – Depends on the person. (P4)
	Repetition	A good program, not sure why it was so repetitive with 9 sections then 2 or 3 repeats in each section. (P2) The tedious repetition of the exercises. (P8)
	Use of iPad	The use of iPad is good if people really understand its use and do not feel too threatened by it. I appreciated learning how to use it – thanks to the people who helped! (P7) …there could be difficulties for some people using an iPad if the people aren't used to them. Could be very confusing and frustrating for folk with dementia. (P4)
	Challenge level	It might help people with minimal memory loss however for people with acute memory loss, this program would be difficult. (P4) I wonder if some of the long-detailed sentences may be too much for some people to learn. I found I skimmed and only remembered the first words of the sentence. (P7)

Implementation	Balancing therapist-led with self-led iPad sessions	Good to get away from a screen and interact with therapist on task… Not all people our age have any experience on these (iPads). In these cases, the use of one-on-one program would be more suitable. (P2) I did better on my own because I did not feel stressed that I might be holding anyone up. (P3) It was pleasant to have supervising (therapists/students) with cheerful attitudes. (P6)
	Programme length	I felt 9 weeks was too long, doing the same tasks 9 times on each subject was too repetitive, not sure what that proves. (P2) Keeping a day a week free for 9 weeks, I did not realise I had such a busy life. (P3)

## Data Availability

The survey data used to support the findings of this study are available from the corresponding author upon request.
